# Addition of Manas barley chromosome arms to the hexaploid wheat genome

**DOI:** 10.1186/s12863-016-0393-2

**Published:** 2016-06-21

**Authors:** Edina Türkösi, András Cseh, Éva Darkó, Márta Molnár-Láng

**Affiliations:** Agricultural Institute, Centre for Agricultural Research, Hungarian Academy of Sciences, H-2462 Martonvásár, P.O. Box 19, Hungary

**Keywords:** Wheat-barley ditelosomic addition lines, Genomic in situ hybridization, Fluorescence in situ hybridization, SSR and STS markers, Morphological parameters, Salt stress response

## Abstract

**Background:**

Cultivated barley belongs to the tertiary genepool of hexaploid wheat. Genes of interest can be transferred from barley into wheat through wide hybridization. The application of wheat-barley introgression lines could provide an excellent tool for the transfer of earliness, favourable amino acid composition, biotic stress resistance, abiotic stress tolerance, or good tillering ability into wheat.

**Results:**

A set of 10 wheat-barley ditelosomic addition lines (2HS, 2HL, 3HS, 3HL, 4HS, 4HL, 6HS, 6HL, 7HS and 7HL) was developed from the progenies of an Asakaze/Manas wheat-barley hybrid produced in Martonvásár, Hungary. The addition lines were selected from self-fertilized plants of the BC_2_F_2_-BC_2_F_4_ generations using genomic in situ hybridization (GISH) and were identified by fluorescence in situ hybridization (FISH) with repetitive DNA probes [HvT01, (GAA)_7_ and centromere-specific (AGGGAG)_4_ probes]. The cytogenetic identification was confirmed using barley arm-specific SSR and STS markers. The ditelosomic additions were propagated in the phytotron and in the field, and morphological parameters (plant height, tillering, length of the main spike, number of seeds/spike and seeds/plant, and spike characteristics) were described. In addition, the salt stress response of the ditelosomic additions was determined.

**Conclusions:**

The six-rowed winter barley cultivar Manas is much better adapted to Central European environmental conditions than the two-rowed spring barley Betzes previously used in wheat-barley crosses. The production of wheat-barley ditelosomic addition lines has a wide range of applications both for breeding (transfer of useful genes to the recipient species) and for basic research (mapping of barley genes, genetic and evolutionary studies and heterologous expression of barley genes in the wheat background).

**Electronic supplementary material:**

The online version of this article (doi:10.1186/s12863-016-0393-2) contains supplementary material, which is available to authorized users.

## Background

Attempts to hybridize wheat with barley were begun more than 100 years ago [1], but the first authentic hybrid was obtained much later by the Danish scientist Kruse [2], who used barley as the female parent [3–5]. The production of the reciprocal hybrid (with wheat as the female parent and barley as the male parent) is more difficult, but this kind of combination is now in the focus of research in this field because of the pistilloidy and male sterility observed in barley × wheat hybrids [6]. The development of the first wheat × barley hybrids was followed by the production of wheat-barley disomic addition lines (2H, 3H, 4H, 5H, 6H and 7H), the first of which arose from crosses between Chinese Spring wheat and the spring barley Betzes [7]. The success of wheat × barley crosses is very dependent on the genotypes used. Previous experience demonstrated that wheat cultivars originating from the Far East have better crossability with related species. Several barley cultivars (Betzes, Igri, Manas and Osnova) were used in wheat-barley crosses in Martonvásár [8]. The Ukrainian six-rowed barley Manas has many useful agronomic characters (e.g. good winter hardiness, abiotic stress tolerance and yield ability) and is well adapted to Central European conditions [9].

The first and as yet only near complete set of hexaploid wheat-barley ditelosomic addition lines was produced by Islam in the 1980’s using a set of monosomic additions of Betzes barley (2n = 2× = 14; HH) chromosomes to hexaploid Chinese Spring wheat (2n = 6× = 42; AABBDD) [10]. Only 12 of the 14 possible ditelosomic addition lines could be developed, as the monosomic addition involving chromosome 1H was self-sterile, due to the presence of the *Shw* sterility gene on the long arm of this chromosome [4]. A ditelosomic addition line involving barley chromosome 1HS was developed later [10]. The wheat-barley chromosome and chromosome arm addition lines are used for assigning genes to chromosomes and chromosome arms and for the characterization of the expression pattern of barley genes in the wheat genetic background. Wheat-barley hybrids can be used for studying the homoeologous relationship between wheat and barley genomes at chromosome level [11, 12]. The barley resistance genes can also be effective in the genetic background of wheat [3], at the same time wheat-barley introgression lines could be an excellent tool for the tranfer of earliness, favourable amino acid composition, biotic stress resistance, salt and drought tolerance, or good tillering ability from barley into wheat [5]. Wheat-barley ditelosomic addition lines, on the other hand, can act as bridging materials for generating wheat-barley translocations, which are more stable than aneuploids.

The aim of this work was to select fertile, genetically stable, wheat-barley ditelosomic addition lines from backcrossed progenies of the Asakaze/Manas wheat-barley hybrid produced earlier in Martonvásár [13, 14]. Barley chromosomes were detected in the wheat background using genomic in situ hybridization (GISH), and identified with fluorescence in situ hybridization (FISH) and molecular (SSR and STS) markers specific for barley chromosome arms. The morphological characters, yield components and salt stress response of the ten lines were also investigated.

## Methods

### Plant material

**Fig. 1:**
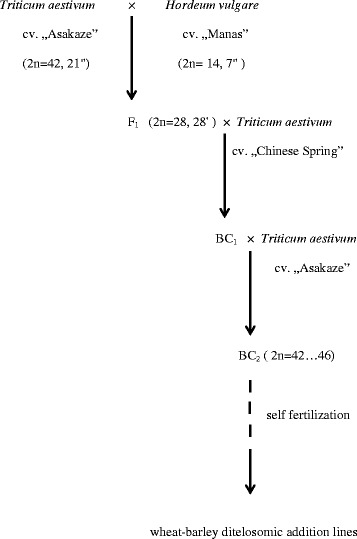
Procedure for isolating barley chromosome ditelosomic addition lines in hexaploid wheat cultivar “Asakaze”

The Japanese facultative wheat Asakaze was used as female parent and the Ukrainian six-rowed winter barley Manas as pollinator to produce a wheat-barley hybrid. The wheat cv. Asakaze and the barley cv. Manas were provided by the Martonvasar Cereal Gene Bank. The hybrid embryo was dissected three weeks after pollination and raised in embryo culture [13]. The hybrid plant had good viability and developed several tillers. As the hybrid was sterile, it was multiplied from young inflorescences in tissue culture. Spikes from 354 regenerant hybrids were pollinated with the wheat cultivars Asakaze, Mv9 kr1 and Chinese Spring, but a BC_1_ progeny was only obtained from the backcross with Chinese Spring. The BC_1_ plant was crossed with wheat cultivar Asakaze and 16 BC_2_ plants were grown to maturity. The presence of barley chromosomes in the wheat background was analysed in the BC_2_ plants with a combination of GISH and molecular markers, as reported earlier by Molnár-Láng et al. [14]. Ten ditelosomic addition lines (2HS, 2HL, 3HS, 3HL, 4HS, 4HL, 6HS, 6HL, 7HS and 7HL) were selected from 860 self-fertilized progenies of the fertile BC_2_ plants (Fig. 1). The morphological traits of the plants were analysed in experiments carried out in phytotron climate chambers (Conviron PGV96) in 2013–2014 and in the field in the Tükrös nursery, Martonvásár, Hungary during the 2014–2015 growing season.

### In situ hybridization

Mitotic chromosome spreads from germinating root tips were prepared as described by Lukaszewski et al. [15]. The ditelosomic addition lines were selected using GISH from the BC_2_F_2_-BC_2_F_4_ generations and identified by FISH using repetitive DNA probes. The GISH experiment was carried out as described by Molnár-Láng et al. [16]. Barley total genomic DNA was labelled with digoxigenin-11-dUTP (Roche Diagnostics, Mannheim, Germany) with a nick translation mix and used as a probe. Unlabelled wheat genomic DNA was used as blocking DNA at a ratio of 35:1. Detection was carried out with anti-digoxigenin-Rhodamine (Roche). The slides were mounted in Vectashield antifade solution (Vector Laboratories, Burlingame, USA) containing 2 μg/mL 4′-6-diamidino-2-phenylindole (DAPI). After rinsing off the GISH hybridization signals in 4 × SSC Tween at room temperature, FISH was carried out using the (GAA)_7_ microsatellite probe [17], the barley subtelomere-specific probe HvT01 [18] and barley centromere-specific sequences (AGGGAG)_4_ [19]. The (GAA)_7_ microsatellite probe was amplified and labelled with biotin-16-dUTP using PCR. The barley subtelomeric sequence HvT01 was labelled combinatorially with 50 % biotin-16-dUTP and 50 % digoxigenin-11-dUTP. The barley centromere-specific sequences (AGGGAG)_4_ were labelled with digoxigenin-11-dUTP. Wheat-specific DNA repetitive probes (pSc119.2, Afa family and pTa71) were used to identify the presence of the entire wheat genome. The repetitive DNA sequences Afa family [20] and pSc119.2 [21] were amplified and labelled with digoxigenin-11-dUTP and biotin-16-dUTP, respectively, using PCR. The 45S rDNA clone pTa71 [22] was labelled with 50 % biotin-16-dUTP and 50 % digoxigenin-11-dUTP by nick translation. Digoxigenin and biotin signals were detected using anti-digoxigenin-Rhodamine Fab fragments and streptavidin-FITC (Roche), respectively. The slides were screened using a Zeiss Axioskop-2 fluorescence microscope equipped with filter sets appropriate for DAPI, FITC, Rhodamine and the simultaneous detection of FITC and Rhodamine (double filter set). Images were captured with a Spot CCD camera (Diagnostic Instruments, Sterling Heights, USA) and processed with Image Pro Plus software (Media Cybernetics, Silver Spring, USA).

### SSR and STS marker analysis

Genomic DNA was extracted from fresh young leaves of wheat cultivar Asakaze, barley cultivar Manas and the ten Asakaze/Manas wheat-barley ditelosomic addition lines using Quick Gene-Mini80 (FujiFilm, Japan) with a QuickGene DNA tissue kit (FujiFilm, Japan) according to the manufacturer’s instructions. The following set of barley chromosome arm-specific SSR markers and gene-specific STS markers was selected: HvCSLF4-2HS [23], Bmag0125-2HL [24], HvLTPPB-3HS [24], HvM60-3HL [25], HvM40-4HS [25], HvM67-4HL [25], Bmac0316-6HS [24], EBmac0806-6HL [24], Bmac0031-7HS [24] and HvCslF6-7HL [23]. All the primer pairs were tested on DNA templates containing: genomic DNA from wheat cultivar Asakaze, barley cultivar Manas and the Asakaze/Manas ditelosomic addition lines. The PCR amplification was performed under the conditions described by Molnár et al. [26]. The primer sequences and annealing temperatures used in this study are presented in Additional file 1. The PCR products were separated with a Fragment Analyzer™ Automated CE System equipped with a 96-Capillary Array Cartridge (Advanced Analytical Technologies, USA). The results were analysed using PROsize v2.0 software (Advanced Analytical Technologies, USA).

### Phenotypic characterization of the plants, and statistical analysis

The Asakaze/Manas wheat-barley ditelosomic addition lines 2HS, 2HL, 3HS, 3HL, 4HS, 4HL, 6HS, 6HL, 7HS and 7HL, the parental genotypes Asakaze, Chinese Spring and Manas and the Asakaze/Manas disomic addition lines were grown in phytotron chambers (Conviron PGV96) in Martonvásár. Vernalization was carried out at 4 °C for 6 weeks, and the plants were grown until tillering under an initial 15 °C day:10 °C night temperature, 12 h light:12 h dark photoperiod, 200 μmol m^−2^s^−1^ light intensity (at pot level) and 75 % relative humidity [27]. The temperature rose by increments of 2 °C after tillering (day length 14 h), stem elongation (16 h illumination) and flowering, and 2 weeks after fertilization. The phenotypic analysis was performed on 10 plants from each genotype. The same genotypes were sown in the field in Martonvásár with 10 seeds in each 1 m row and a row distance of 15 cm. Ten plants were randomly selected from each genotype for analysis. Plant height and tillering were determined immediately before harvest. The length of the main spike, spikelets/main spike, seeds/main spike and seeds/plant were measured after harvest.

The morphological traits of the ditelosomic addition lines were compared with those of the Asakaze and Chinese Spring wheat genotypes using Student’s t test for paired data at the *P* = 0.05 significance level.

### Evaluation of the flowering date of Asakaze/Manas ditelosomic addition lines

The flowering time of 10 plants was recorded for each genotype in the field experiment. Statistical analysis was performed using Student’s t test for paired data on the flowering date and significant differences at the *P* = 0.05 significance level.

### Evaluation of the salt stress response of Asakaze/Manas ditelosomic addition lines

The salt stress response of seedlings of Asakaze/Manas ditelosomic addition lines was screened and compared to that of wheat and barley. The other wheat crossing parent Chinese Spring was not used in these investigations, as it had similar or lower salt tolerance than Asakaze, as demonstrated in earlier investigations [28]. In the germination test, 3× 20 seeds of each genotype per treatment were surface-sterilized in 10 % sodium hypochlorite for 15 min, rinsed twice in distilled water and germinated on wet filter paper containing 0, 100, 200 or 250 mM NaCl in Petri dishes for 3 days at a temperature of 25 °C. The percentage of germinated seeds and the length and weight of roots and coleoptiles were determined.

## Results

### Selection, identification and morphological characterization of the wheat-barley ditelosomic addition lines

**Fig. 2:**
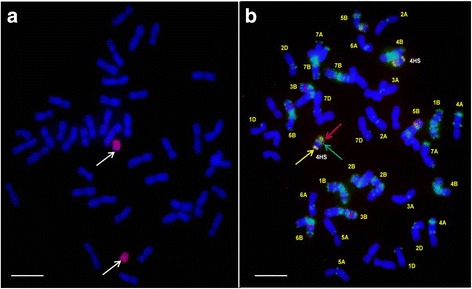
**a** Genomic in situ hybridization on mitotic chromosomes of the 4HS Asakaze/Manas wheat-barley ditelosomic addition. The chromosomes were counterstained with DAPI (*blue*). Barley total genomic DNA is visualized in red (*arrows*). **b** Fluorescence in situ hybridization on mitotic chromosomes of the Asakaze/Manas wheat-barley 4HS ditelosomic addition with DNA repetitive probes: barley centromere-specific probe (*red* and *red arrow*), (GAA)_7_ microsatellite probe (*green* and *green arrow*) and HvT01 (*yellow* and *yellow arrow*). Scale bar 10 = μm

**Fig. 3:**
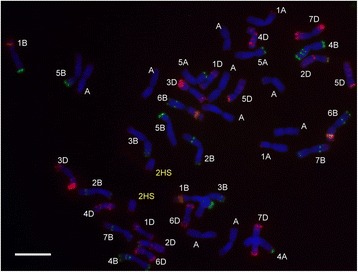
Fluorescence in situ hybridization on mitotic chromosomes of the Asakaze/Manas wheat-barley 2HS ditelosomic addition with DNA repetitive probes: pSc119.2 (*green*), Afa family (*red*) and pTa71 (*orange*). Scale bar 10 = μm

All the barley chromosomes were present in one or more of the 16 BC_2_ plants originating from the Asakaze × Manas hybrid with the exception of the complete 5H, though 5HS was present in one plant. It was not possible to select lines with either the 1H or 5HS additions, because the BC_2_ plants carrying these barley chromosomes set no seed [9]. Ten wheat-barley ditelosomic additions were selected from the progenies of the BC_2_ plants. These lines were 2HS, 2HL, 3HS, 3HL, 4HS, 4HL, 6HS, 6HL, 7HS and 7HL. The presence of the barley telocentric chromosomes in the wheat background was detected using GISH (Fig. 2a). The telocentric barley chromosomes were identified using FISH (Fig. 2b) and barley-arm specific SSR and STS markers. The barley subtelomere-specific probe HvT01 and barley centromere-specific sequences (AGGGAG)_4_ were used to demonstrate that the entire barley chromosome arm was present in the relevant ditelosomic addition line. The presence of 42 wheat chromosomes was detected with wheat-specific DNA repetitive FISH probes (Fig. 3). The wheat-barley ditelosomic addition lines were developed from 860 progenies of selfed BC_2_ plants, mainly from monosomic addition lines, between 2010 and 2013. Thirty percent of these plants (260) were homozygous telosomic plants, while the barley chromosomes were eliminated from 46 % (395) of the plants. Monotelosomic additions, Robertsonian translocations, mono- and disomic additions and isochromosomes were found among the lines investigated (Table 1).

**Table 1:** The number of seeds examined for the development of each wheat-barley ditelosomic addition line

Genotypes	Barley chromosomes detected in the seeds analysed				
	2 telocentrics	1 telocentric	Others	Barley chromosomes eliminated	Number of seeds examined
2HS	17	6	0	14	37
2HL	19	4	6	17	46
3HS	51	19	1	38	109
3HL	12	24	3	86	125
4HS	33	45	1	115	194
4HL	23	10	0	16	49
6HS	13	19	25	56	113
6HL	29	13	0	6	48
7HS	10	6	0	8	24
7HL	53	17	6	39	115
∑	260	163	42	395	860

**Fig. 4:**
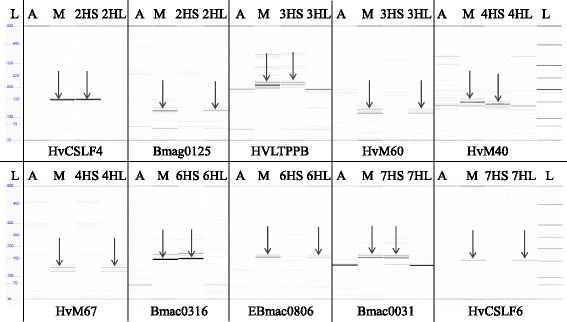
Capillary gel electrophoresis pattern of the ditelosomic addition lines using HvCSLF4-2HS, Bmag0125-2HL, HvLTPPB-3HS, HvM60-3HL, HvM40-4HS, HvM67-4HL, Bmac0316-6HS, EBmac0806-6HL, Bmac0031-7HS and HvCSLF6-7HL barley chromosome arm-specific markers on the following DNA templates: Asakaze wheat (A), Manas barley (M), A/M ditelosomic addition lines (2HS, 2HL; 3HS, 3HL; 4HS, 4HL; 6HS, 6HL; 7HS, 7HL). Barley chromosome arm-specific bands are indicated by arrows. A 50-bp DNA ladder (L) was used to estimate fragment size

**Fig. 5:**
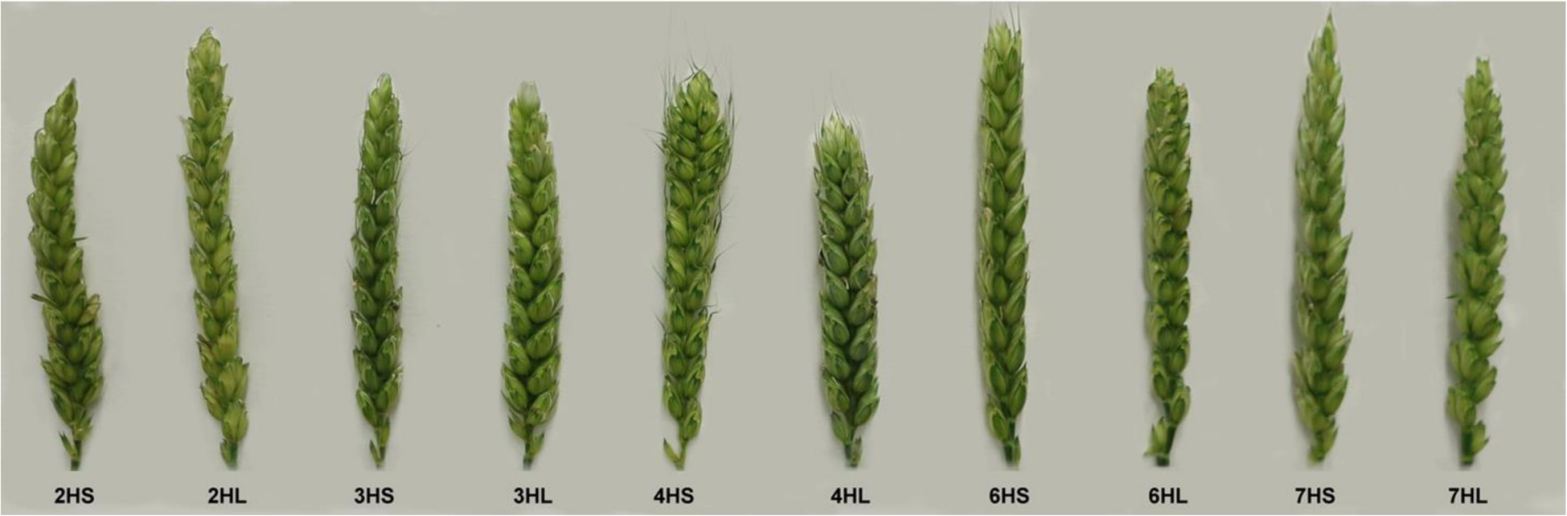
Spike morphology of the ten Asakaze/Manas ditelosomic addition lines (typical spikes collected from the Tükrös nursery, Martonvásár, May 2015)

The 2HS and 2HL ditelosomic addition lines were selected from a 2H monosomic addition. Some of its progenies carried telocentric chromosomes, which were assumed to be 2HS or 2HL telocentrics. The barley telocentric chromosome pair was detected in the wheat background using GISH. The cytological analysis was followed by the identification of the alien chromatin using the HvCSLF4 STS marker to identify the 2HS arm and the Bmag0125 SSR marker for the 2HL chromosome arm (Fig. 4). The 2HS addition line had awnless, tapered spikes with a specific curvature and hard culm, while the spike of the 2HL addition plants was awnless with a laxed structure (Fig. 5). In the phytotron experiments the two lines showed significantly better tillering and number of seeds/plant than the Asakaze wheat genotype (Additional file 2).

The 3HS chromosome addition was selected from the progenies of a 3H monosomic addition, while the 3HL was identified among the descendants of a 3H disomic addition. After detecting the barley chromatin using GISH, the 3HS and 3HL additions were identified with the help of the HvLPTTB and HvM60 SSR markers, respectively (Fig. 4). The 3HS line had laxed spikes with awnstubs, while the 3HL line had short, awnless spikes with a denser structure (Fig. 5). In both phytotron and field experiments the 3HL line had one of the shortest spikes of all the ditelosomic additions developed, yet the 1000-kernel weight calculated for the field experiment was better than that of almost all the other ditelosomic and disomic addition lines (Additional files 2 and 3).

The 4HS and 4HL ditelosomic addition lines were derived from the 4H monosomic addition line. Telocentric barley chromosomes were identified in the wheat background on the mitotic chromosome spreads of its progenies using GISH. The cytological identification was confirmed using SSR markers specific for the 4HS (HvM40) and 4HL (HvM67) barley chromosome arms (Fig. 4). The 4HS telocentric chromosome was eliminated from the analysed plants with the highest frequency (Table 1), while the homozygous 4HS ditelosomic addition was quite stable. The 4HS line had flared spikes with awnstubs, while 4HL had shorter, dense spikes also possessing awnstubs (Fig. 5). The 4HL ditelosomic addition plants were the shortest in the series of ten ditelosomic addition lines; the plant height was significantly lower than that of the wheat genotypes both in the phytotron and in the field. The fertility of both the 4HS and 4HL lines was better than that of the other eight ditelosomic addition lines, both in the phytotron and in the field experiment (Additional files 2 and 3).

A double monosomic addition line carrying the 6H and 7H barley chromosomes was the parental genotype for the 6HS, 6HL and some of the 7HL wheat-barley ditelosomic additions. The telocentrics of the 6H chromosome were transmitted with higher frequency than that of the 7H. The 7HS arm was not detected in the progenies of these plants. The arms of the 6H chromosome were detected using GISH and identified with barley arm-specific SSR markers. The Bmac0136 marker revealed the presence of the 6HS barley chromosome arm, while the EBmac0806 confirmed the presence of 6HL (Fig. 4). The 6HS line had long spikes with apical awnstubs, while the 6HL spike was awnless (Fig. 5).

The 7HS and 7HL addition lines were selected from the the progenies of the 7H monosomic addition. The cytogenetic identification of the barley chromatin in the wheat background was followed by SSR and STS marker analysis, using the Bmac0031 SSR and HvCSLF6 STS markers, which distinguished the 7HS and 7HL barley chromosome arms, respectively (Fig. 4). Though the 7HL ditelosomic adition line was stable, more than two telocentric chromosomes were detected in some of the mitotic chromosome spreads of homozygous plants. As GISH did not reveal the barley origin of these chromosomes, FISH was applied on the same chromosome preparations after washing off the GISH signals. Wheat-specific FISH probes (pSc119.2, Afa family and pTa71) were used to identify the fragmented wheat chromosome. In all the preparations analysed this was found to be the 4B chromosome. Both the 7HS and 7HL additions had awnless spikes that were not very dense, but the 7HS spike was much longer (Fig. 5). In plants carrying the fragmented 4B wheat chromosome, the spikes of the 7HL addition were much shorter and had very low fertility. The fertility of the 7HS line was also low, as its spikes had many sterile spikelets on the apical part.

During the propagation of the lines the stability of the lines was also investigated in the progenies of plants homozygous for the presence of the barley telocentric chromosomes. The stability of all ten ditelosomic adition lines was higher than 50 % and varied from 57.14 % for the 2HS addition (of 28 progenies of ditelosomics, 16 were also ditelosomics) and 100 % for 2HL (all 26 progenies of plants homozygous for the barley telocentrics were also ditelosomics) (Table 2).

**Table 2:** Stability of the Asakaze/Manas ditelosomic addition lines, investigated in the progenies of homozygous ditelosomic plants

Genotypes	Number of progenies of ditelosomic analysed	Ditelosomic plants	Rate of ditelosomics among progenies
2HS	28	16	57.14 %
2HL	26	26	100 %
3HS	36	30	83.33 %
3HL	30	19	63.33 %
4HS	45	38	84.44 %
4HL	20	19	95 %
6HS	44	28	63.63 %
6HL	36	28	77.77 %
7HS	23	18	78.26 %
7HL	81	73	90.12 %

### Evaluation of flowering time of Asakaze/Manas ditelosomic addition lines in the field experiment

**Fig. 6:**
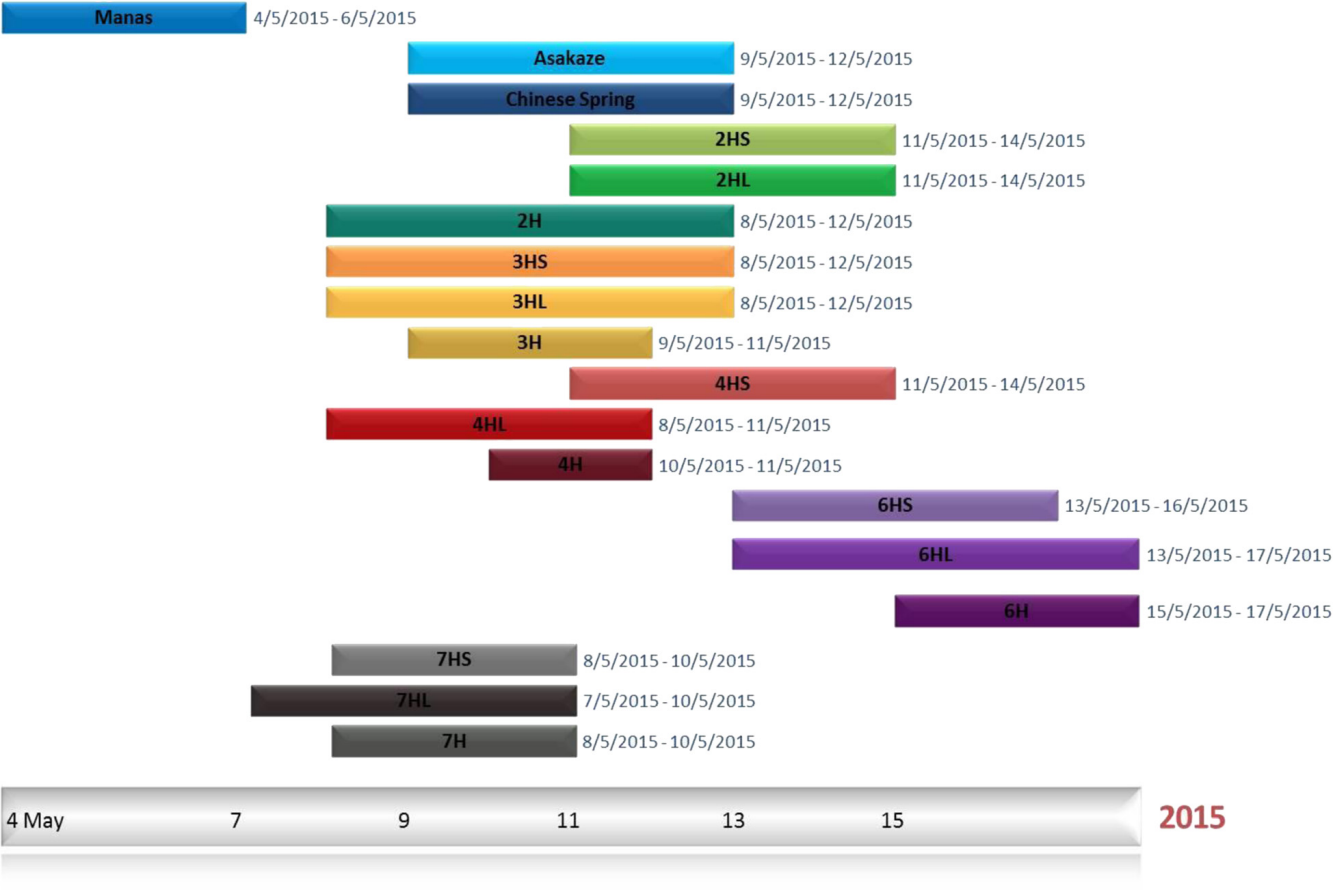
Evaluation of the flowering time of Asakaze/Manas ditelosomic addition lines compared to that of the parental genotypes (Manas, Asakaze and Chinese Spring) and Asakaze/Manas disomic addition lines. Data were recorded for the main spikes of 10 randomly selected plants from each genotype in the field experiment (Tükrös nursery, Martonvásár, May 2015)

The flowering time of the ditelosomic addition lines was evaluated in the field experiment together with that of the Asakaze/Manas disomic 2H, 3H, 4H, 6H and 7H addition lines, the Asakaze and Chinese Spring wheat cultivars and the Manas barley cultivar. The earliest flowering genotype was Manas, where all the main spikes of the ten assigned plants flowered between 4 and 6 May. The earliest addition line was 7HL, which began flowering on 7 May, followed immediately on 8 May by the Asakaze wheat cultivar and the 2H, 3HS, 3HL, 4HL, 7HS and 7H addition lines, though the flowering interval was longer for most of these lines than for the 7HL addition line. The latest flowering lines were 6HS and 6HL on 13 May and 6H on 15 May (Fig. 6, Table 3).

**Table 3:** Average flowering date of the genotype analysed (No. of days from 1st January 2015)

Genotype	2HS	2HL	2H	3HS	3HL	3H	4HS	4HL	4H	6HS	6HL	6H	7HS	7HL	7H	Asakaze	CS	Manas
Average flowering date	132.4^a^,^b^,^c^	131.6^a^,^b^,^c^	129.8	129.2^b^	130.3^a^	130^a^	132.5^a^,^b^	128.8^b^	130.8^a^	134.2^a^,^b^	134.5^a^,^b^	136^a^,^b^	129.4^b^	128.7^b^	128.3^b^	129	130.4	124.7
Standard deviation	1.35	1.26	1.13	1.14	1.34	0.67	0.97	1.31	0.42	1.14	1.59	0.81	0.70	1.16	0.67	0.81	1.07	0.82

^a^Significantly different from the Asakaze wheat cultivar at the *P* < 0.05 significance level

^b^Significantly different from the Chinese Spring wheat cultivar at the *P* < 0.05 significance level

^c^Significantly different from the 2H line at the *P* < 0.05 significance level

### Evaluation of the salt stress response of Asakaze/Manas ditelosomic addition lines

**Fig. 7:**
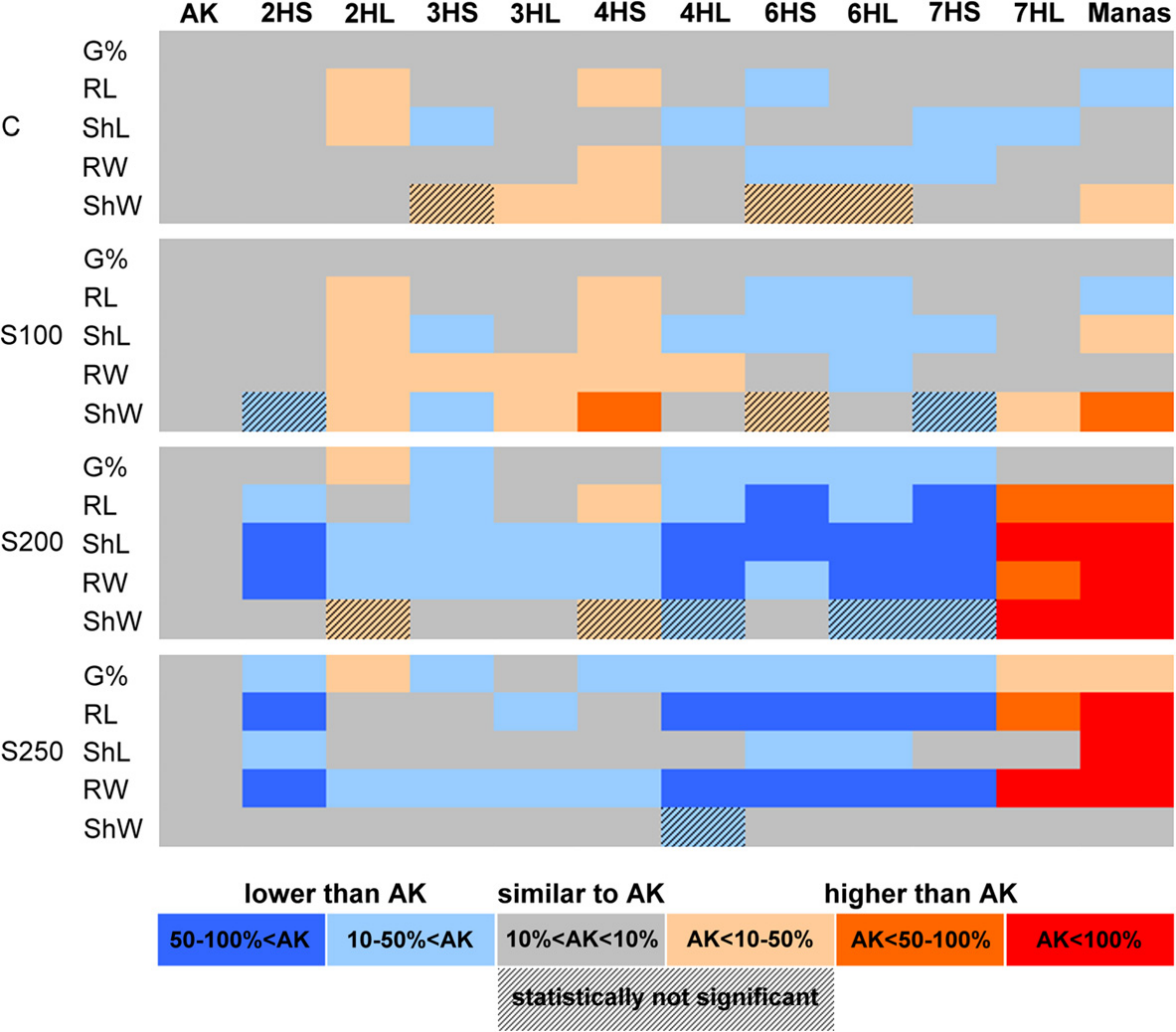
Comparison of the germination properties (germination rate, G%, root length, RL, shoot length, ShL, root weight, RW, shoot weight and ShW) of seedlings of wheat cultivar Asakaze (A), Asakaze/Manas ditelosomic addition lines and barley cultivar Manas in the control (C, without salt treatment) and under salt stress conditions induced by 100 mM (S100), 200 mM (S200) and 250 mM (S250) NaCl treatment. Grey blocks demonstrate that the values are similar (within 10 %) to those of wheat Asakaze. Reddish and blue blocks indicate higher and lower values than in wheat Asakaze, respectively. The absolute values are found Additional file 4

Germination tests were used for evaluating the salt stress response of Asakaze/Manas ditelosomic addition lines. The results were compared with those of the wheat parent Asakaze and the barley parent Manas and the differences were presented in Fig. 7. Without salt treatment, the genotypes showed only slight variability. The ditelosomic addition lines 2HL and 4HS had better growth vigour than Asakaze, while lines 6HS, 6HL and 7HS had poorer vigour. The 2HL addition line exhibited higher root and shoot length than wheat Asakaze. The root growth (both length and weight) was also higher in ditelosomic line 4HS than in Asakaze. These differences were more pronounced under mild salt stress (100 mM salt treatment), but disappeared when higher salt concentrations (200 and 250 mM) were applied (Fig. 7). The salt-induced decrease in germination rate and root and shoot growth (both length and weight) was considerable in the case of the 4HL, 6HS, 6HL and 7HS ditelosomic lines. Inversely, higher growth potential was retained under salt stress conditions in ditelosomic line 7HL and Manas, which had higher root and shoot lengths and weight data than Asakaze. However, it should also be mentioned that salt stress inhibited shoot growth more intensively than root growth. The severe (200 and 250 mM) salt treatment led to 1–2 mm shoot primordia, which made it impossible to discriminate between the genotypes, as indicated in Additional file 4.

In previous experiments several wheat/barley addition and translocation lines carrying chromosomes from different wheat and barley cultivars were studied for aluminium and salt tolerance [29, 30]. A comparison of the salt stress responses of Asakaze/Manas wheat–barley disomic addition lines (2H, 3H, 3HS, 4H, 6H, 7H and 7HL) to those of the parental genotypes (Asakaze, Manas and Chinese Spring) revealed that the disomic addition line 7H and the ditelosomic line 7HL exhibited higher salt tolerance both during germination and in the early developmental stages than the wheat parents, among which Asakaze had higher salt tolerance than Chinese Spring [30]. The present experiment using a set of ditelosomic lines (2HS, 2HL, 3HS, 3HL, 4HS, 4HL, 6HS, 6HL, 7HS and 7HL) confirmed the previous results. The ditelosomic line 7HS showed a higher reduction in growth under salt stress conditions than the wheat parent Asakaze, and the ditelosomic line 7HL and the barley parent Manas exhibited higher salt-tolerance at germination than Asakaze. These results suggest that lines carrying the long arm of the 7H barley chromosome in the wheat background could be used as genetic material for improving the salt tolerance of wheat. Higher root and shoot growth was also observed in the ditelosomic lines 2HL and 4HS than in wheat Asakaze during germination under control and mild salt stress conditions, but this improved vigour disappeared under severe salt stress, indicating that the intense growth was not related to the salt stress response. Intense root growth was also observed previously in 4H disomic addition lines [29], and the present results demonstrate that this is associated with the 4HS chromosome arm.

## Discussion

The present paper reports the development of a new set of wheat-barley ditelosomic addition lines (2HS, 2HL, 3HS, 3HL, 4HS, 4HL, 6HS, 6HL, 7HS and 7HL) obtained by incorporating the chromosomes of the six-rowed Ukrainian winter barley cultivar Manas into the facultative Japanese wheat cultivar Asakaze. The 5HS and 5HL ditelosomics could not be selected from the Asakaze/Manas combination, as the 5H chromosome was eliminated most frequently from the backcross progenies and the plant carrying the arms of this chromosome was sterile [14]. Each ditelosomic addition line developed contained the full complement of wheat chromosomes and a single telocentric chromosome pair from barley, though in some of the plants analysed wheat chromosome breakages or chromosome duplications were observed. Barley telocentric chromosomes may result from the misdivision of barley univalents during meiosis. The first and as yet only near complete set of wheat-barley ditelosomics, including 13 of the 14 possible ditelosomic addition lines (except 1HL) was produced by Islam in the 80’s through the addition of barley chromosomes from the Betzes barley cultivar to the Chinese Spring wheat cultivar. These lines were characterized on the basis of their morphological, physiological and biochemical features and the N-banding analysis of the chromosomes [10]. The newly developed Asakaze-Manas wheat-barley ditelosomic addition lines were identified using GISH, FISH and molecular marker analysis, while the morphological traits and yield components were studied in phytotron and field experiments.

Manas is a winter barley cultivar with good winter hardiness making it better adapted to Central European climatic conditions than Betzes. It also has favourable agronomic traits, including good yielding ability [9] and better salt and aluminium tolerance than the other two barley genotypes previously used to develop addition lines [28–30]. Wheat-alien ditelosomic additions have also been developed using other cultivated and wild relatives of wheat, including various cultivars of rye, *Aegilops* species, *Thinopyrum ponticum*, *Elymus* species, *Leymus racemosus* and *Hordeum chilense*. These ditelosomic addition lines can be utilized for a wide range of purposes [31–40].

The development of Chinese Spring/Betzes wheat-barley ditelosomic additon lines opened the door for the flow sorting of barley telocentric chromosomes at high purity [41], making it possible to construct chromosome arm-specific DNA libraries and to perform the cytogenetic mapping needed for the development of physical contig maps [42]. Mayer et al. [43] reported the use of flow-sorted barley chromosome 1H and chromosome arms 2HS to7HL to construct a high resolution sequence-based gene map containing an estimated 86 % of the genes in the barley genome. It also proved possible to strech flow-sorted plant chromosomes longitudinally, thus increasing the physical resolution of maps constructed using FISH [44].

The most widespread wheat-alien introgression is the 1BL.1RS wheat-rye tranlocation where the 1RS chromosome arm carries resistance genes to biotic and abiotic stresses, and genes affecting yield potential or protein content. The 1BL.1RS translocation is present in several hundreds of wheat cultivars, and has therefore received great attention from researchers and breeders. Flow-sorted 1RS chromosomes from the 1RS wheat-rye (Chinese Spring-Imperial) ditelosomic addition line were used to construct BAC libraries specific for 1RS rye chromosome. Chromosome arm-specific BAC libraries make it possible the high-resolution analysis of a particular region of complex plant genomes and developing molecular markers for these regions [45].

With the help of the Chinese Spring-*Aegilops geniculata* 5M^g^ ditelosomic addition line, the arm of an *Aegilops* chromosome, was successfully flow-sorted for the first time and sequenced using Illumina technology. Next-generation sequencing offers a cheap way to develop sequence-based markers for the molecular analysis of *Aegilops* chromosomes [46].

The Asakaze/Manas wheat-barley ditelosomic addition lines were compared with the Asakaze/Manas disomic addition lines, previously selected from the same wheat-barley cross [9]. As aneuploids, disomic and ditelosomic additions exhibit a certain degree of instability which necessitates regular cytological analysis. Based on the experiments carried out to date it can be concluded that the Asakaze/Manas ditelosomic addition lines are more stable than the disomic addition lines. The 3H disomic addition, in particular, showed an unexpectedly high level of chromosome instability in comparison with that developed from Mv9kr1 and Igri [9, 47], while both 3HS and 3HL were more stable during the propagation of the ditelosomic addition lines (Table 2). The transmission of the alien chromosomes was more reliable when single alien chromosomes were replaced by their telocentrics [48].

**Fig. 8:**
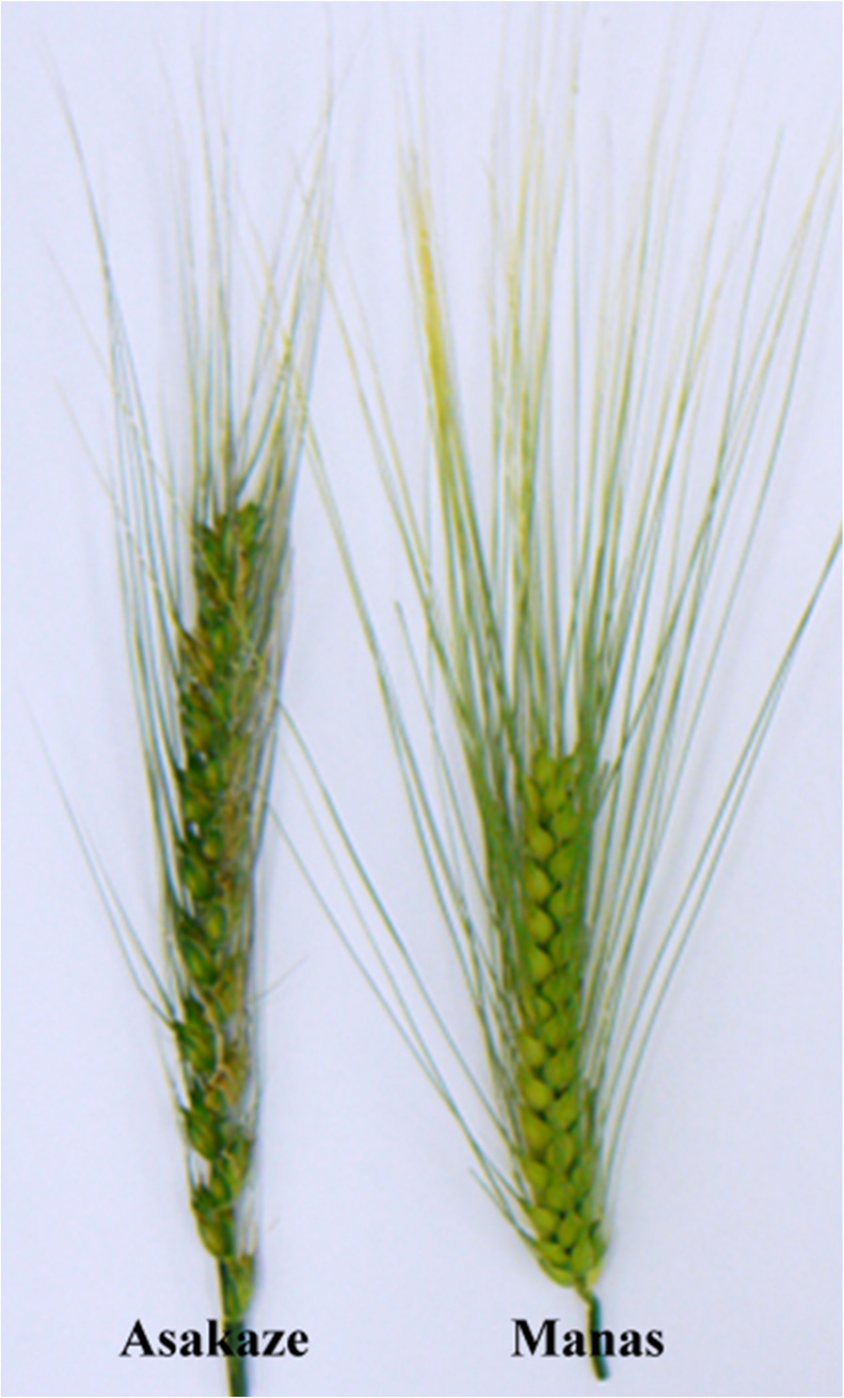
Spike morphology of wheat cultivar Asakaze and barley cultivar Manas (typical spikes collected from the Tükrös nursery, Martonvásár, May 2015)

Differences were revealed in the plant and spike morphology and in the fertility of ditelosomic additions compared with those of disomic addition lines. The 4HL addition was the shortest of all the ditelosomic addition lines and the plants were also significantly shorter than the wheat parental genotypes, both in the phytotron and in the field. The 6HS, 6HL and 7HS additions had the longest spikes among the ditelosomic additions, while 4HS and 4HL had significantly shorter spikes but better fertility than the wheat parental genotypes (Figs. 5 and 8). This was especially true of 4HL, which had spikes with a dense structure and a significantly higher number of seeds/spike than Asakaze and Chinese Spring in the experiment carried out in the Tükrös nursery. While the 7H disomic addition line had the lowest fertility ([9] and data presented in this work) and that of 7HL was the lowest of all the ditelosomics, 7HS exhibited higher fertility in the field. The addition line carrying the 7HL chromosome arm was the earliest flowering of the addition lines, flowering later than Manas but earlier than the wheat genotypes, whereas the latest flowering lines were those carrying the entire 6H or the telocentric 6HS and 6HL chromosomes. These results are in agreement with data previously presented by Molnár-Láng et al. [49]. In the field experiment the 2H disomic addition line flowered significantly earlier than lines carrying the short or long arm of this barley chromosome. The major photoperiod sensitivity locus *Ppd-H1* located on 2HS, results in accelerated plant development under a long photoperiod in barley [50, 51]. Winter barleys require vernalization and flowering is usually promoted by long days. Dominant alleles at *Ppd-H1* confer early flowering under long days, but have no effect under short days. The 2H chromosome also contains many important genes for barley development and adaptation, such as row-type *vrs1* [52], earliness per se (*eps2S*) [53, 54] and early maturity *Eam1* [55]. Further genetic and genomic studies will be required to determine how these genes located on the short or long arms of chromosome 2H function separately in a wheat background.

Introgression lines are useful for investigating heterologous expression of genes from alien chromosomes in the wheat genetic background [56]. In previous experiments several wheat/barley addition and translocation lines carrying chromosomes from different wheat and barley cultivars were studied for aluminium and salt tolerance [29, 30]. A comparison of the salt stress responses of Asakaze/Manas wheat–barley disomic addition lines (2H, 3H, 3HS, 4H, 6H, 7H and 7HL) to those of the parental genotypes (Asakaze, Chinese Spring and Manas) revealed that the disomic addition line 7H and the ditelosomic line 7HL exhibited higher salt tolerance both during germination and in the early developmental stages than the wheat parents, among which Asakaze had higher salt tolerance than Chinese Spring [28]. The present experiment, using a set of ditelosomic lines (2HS, 2HL, 3HS, 3HL, 4HS, 4HL, 6HS, 6HL, 7HS and 7HL), confirmed the previous results. The ditelosomic line 7HS exhibited a greater reduction in growth under salt stress conditions than the wheat parent Asakaze, while ditelosomic line 7HL and the barley parent Manas had better salt-tolerance in the germination stage than Asakaze. These results suggest that lines carrying the long arm of 7H barley chromosome in the wheat background could be useful genetic material for improving the salt tolerance of wheat. Higher root and shoot growth was also observed in the ditelosomic lines 2HL and 4HS than in Asakaze during germination under control and mild salt stress conditions, but this improved vigour disappeared under severe salt stress, indicating that the intense growth was not related to the salt stress response. Intense root growth was also observed previously in 4H disomic addition lines [29], and the present results demonstrated that this is associated with the 4HS chromosome arm.

Disomic and ditelosomic addition lines can also be used for the development of wheat-alien translocation lines, which are more stable than additions. The progenies of a monosomic 7H addition line originating from the Asakaze × Manas hybrid were screened for the presence of barley chromatin, revealing the presence of a 4BS.7HL wheat-barley Robertsonian translocation [57]. An increased (1,3;1,4)-β-D-glucan level was detected in the translocation line, demonstrating that the *HvCSLF6* gene, present on the centromeric region of the 7HL chromosome arm and responsible for β-glucan production, was expressed in the genetic background of hexaploid wheat. Based on this knowledge, a compensating 7BS.7HL wheat-barley Robertsonian translocation has been developed, as homoeologous chromosomes of barley are better able to compensate the loss of a certain wheat chromosome. The 7BS.7HL translocation line was developed from a cross between the 7B monosomic Rannaya wheat cultivar and the 7H Asakaze/Manas disomic addition. Plants carrying 42 chromosomes (monosomic for the 7B wheat chromosome and for the 7H barley chromosome) were selected from the F_1_ generation using GISH. The F_1_ plants were self-fertilized and the presence of the short or long arm of the 7H barley chromosome was detected using 7H arm-specific molecular markers in the F_2_ generation. Six plants carrying a monosomic 7BS.7HL centric fusion were selected from the F_3_ generation and were selfed. In the F_4_ generation three plants with 42 chromosomes were identified as disomic 7BS.7HL translocations using GISH, FISH and molecular markers [58]. The (1,3;1,4)-β-D-glucan content of the seeds will be determined after the propagation of these lines. If the *HvCslF6* gene is expressed in this background, the 7BS.7HL Robertsonian translocation stock could be of potential importance for the manipulation of wheat (1,3;1,4)-β-D-glucan levels.

## Conclusions

The present study reports the development of a set of wheat-barley ditelosomic addition lines and ways in which these lines could be used in breeding programmes and for basic research. Several of the agronomic characters of the Manas barley cultivar are better than those of the Betzes cultivar previously used for the development of wheat-barley ditelosomic addition lines. The analysis of the salt tolerance of lines carrying the 7HL chromosome arm is the first step in exploitation of these traits. However, if this genetic material is to be used in wheat breeding, it will be necessary to develop wheat-barley translocation lines carrying only a small fragment of the barley chromatin.

## Abbreviations

DAPI, 4′-6-diamidino-2-phenylindole; FISH, fluorescence in situ hybridization; GISH, genomic in situ hybridization; PCR, polymerase chain reaction; SSC, saline sodium citrate; SSR, simple sequence repeat; STS, sequence tagged site

## Additional files

Additional file 1: STS and SSR markers used in the present study, the primer sequences, the annealing temperature and the size of PCR products. (DOCX 16 kb) Additional file 2: Morphological traits of the Asakaze/Manas wheat-barley ditelosomic addition lines, the parental wheat cultivars and Asakaze/Manas disomic addition lines in experiments carried out in the Martonvásár phytotron between December 2013 and March 2014. #: significantly different from Asakaze, ##: significantly different from Chinese Spring, ###: significantly different from Asakaze and Chinese Spring at *P* = 0.05. (DOCX 17 kb) Additional file 3: Morphological traits of the Asakaze/Manas wheat-barley ditelosomic adittion lines, the parental wheat cultivars and Asakaze/Manas disomic addition lines in the Martonvásár nursery during the 2014–2015 growing season. #: significantly different from Asakaze, ##: significantly different from Chinese Spring, ###: significantly different from Asakaze and Chinese Spring at *P* = 0.05. (DOCX 19 kb) Additional file 4: Effect of salt stress on the germination properties of seedlings of Asakaze/Manas ditelosomic addition lines, the wheat parent Asakaze and the barley parent Manas. The data are mean values ± standard deviation for each treatment. Significant differences were determined using Tukey’s post hoc test and different letters indicate significant differences between the genotypes and treatments at the *P* ≤ 0.05 level. “-” indicates that the drastic reduction caused by salt treatment prevented statistical analysis and the comparison of the genotypes. (DOC 117 kb)

## References

[1] Farrer W (1904). Some notes on the wheat “Bobs”; its peculiarities, economic value, and origin. Agric Gaz NSW.

[2] Kruse A (1973). Hordeum × Triticum hybrids. Hereditas.

[3] Islam AKMR, Shepherd KW, Bajaj YP (1990). Incorporation of barley chromosomes into wheat. Biotechnology in agriculture and forestry wheat.

[4] Islam AKMR, Shepherd KW (2000). Isolation of a fertile wheat-barley addition line carrying the entire barley chromosome 1H. Euphytica.

[5] Molnár-Láng M, Linc G, Szakács É (2014). Wheat–barley hybridization: The last 40 years. Euphytica.

[6] Islam AKMR, Shepherd KW, Sparrow DHB (1981). Isolation and characterization of euplasmic wheat-barley chromosome addition lines. Heredity.

[7] Islam AKMR, Shepherd KW, Sparrow DH. Production and characterization of wheat-barley addition lines. In: Proceedings of 5th International Wheat Genetic Symposium. Ramanujam S. New Delhi ed. India: Science Publishers Inc.; 1978. p. 365–71.

[8] Molnár-Láng M, Linc G, Sutka J (1999). Production and molecular cytogenetic identification of wheat-barley hybrids and translocations. J Plant Biotechnol.

[9] Molnár-Láng M, Kruppa K, Cseh A, Bucsi J, Linc G (2012). Identification and phenotypic description of new wheat – six-rowed winter barley disomic additions. Genome.

[10] Islam AKMR. Ditelosomic additions of barley chromosomes to wheat. In: Proceedindgs of 6th international wheat Genetics Symposium. Sakamoto S. ed. Kyoto, Japan: Kyoto University Press; 1983. p. 233–8.

[11] Cho S, Garvin DF, Muehlbauer GJ (2006). Transcriptome analysis and physical mapping of barley genes in wheat–barley chromosome addition lines. Genetics.

[12] Bilgic H, Cho S, Garvin DF, Muehlbauer GJ (2007). Mapping barley genes to chromosome arms by transcript profiling of wheat-barley ditelosomic chromosome addition lines. Genome.

[13] Molnár-Láng M, Linc G, Logojan A, Sutka J (2000). Production and meiotic pairing behaviour of new hybrids of winter wheat (*Triticum aestivum*) × winter barley (*Hordeum vulgare*). Genome.

[14] Molnár-Láng M, Novotny C, Linc G, Nagy ED (2005). Changes in the meiotic pairing behaviour of a winter wheat-winter barley hybrid maintained for a long term in tissue culture, and tracing the barley chromatin in the progeny using GISH and SSR markers. Plant Breeding.

[15] Lukaszewski AJ, Rybka K, Korzun V, Malyshev SV, Lapinski B, Whitkus R (2004). Genetic and physical mapping of homoeologous recombination points involving wheat chromosome 2B and rye chromosome 2R. Genome.

[16] Molnár-Láng M, Cseh A, Szakács É, Molnár I (2010). Development of a wheat genotype combining the recessive crossability alleles *kr1kr1kr2kr2* and the 1BL.1RS translocation, for the rapid enrichment of 1RS with new allelic variation. Theor Appl Genet.

[17] Pedersen C, Linde-Laursen I (1994). Chromosomal locations of four minor rDNA loci and a marker microsatellite sequence in barley. Chromosome Res.

[18] Schubert I, Shi F, Fuchs J, Endo TR (1998). An efficient screening for terminal deletions and translocations of barley chromosomes added to common wheat. Plant J.

[19] Hudakova S, Michalek W, Presting GG, ten Hoopen R, dos Santos K, Jasencakova Z, Schubert I (2001). Sequence organization of barley centromeres. Nucleic Acids Res.

[20] Nagaki K, Tsujimoto H, Isono K, Sasakuma T (1995). Molecular characterization of a tandem repeat, Afa family, and its distribution among Triticeae. Genome.

[21] Bedbrook JR, Jones J, O’Dell M, Thompson RD, Flavell RB. A molecular description of telomeric heterochromatin in secale species. Cell. 1980;19:545–60. 10.1016/0092-8674(80)90529-2 6244112

[22] Gerlach WL, Bedbrook JR (1979). Cloning and characterization of ribosomal RNA genes from wheat and barley. Nucleic Acids Res.

[23] Burton RA, Jobling SA, Harvey AJ, Shirley NJ, Mather DE, Bacic A, Fincher GB (2008). The genetics and transcriptional profiles of the cellulose synthase-like *HvCslF* gene family in barley. Plant Physiol.

[24] Ramsay L, Macaulay M, Degli IS, MacLean K, Cardle L, Fuller J (2000). A Simple Sequence Repeat-Based Linkage Map of Barley. Genetics.

[25] Liu ZW, Biyashev RM, Maroof MA (1996). Development of simple sequence repeat DNA markers and their integration into a barley linkage map. Theor Appl Genet.

[26] Molnár I, Kubaláková M, Šimková H, Farkas A, Cseh A, Megyeri M, Vrána J, Molnár-Láng M, Doležel J (2014). Flow cytometric chromosome sorting from diploid progenitors of bread wheat, *T. urartu*, *Ae. speltoides* and *Ae. tauschii*. Theor Appl Genet.

[27] Tischner T, Kőszegi B, Veisz O (1997). Climatic programms used in the Martonvasar phytotron most frequently in recent years. Acta Agron Hung.

[28] Darkó É, Janda T, Majláth I, Szopkó D, Dulai S, Molnár I, Türkösi E, Molnár-Láng M (2015). Salt stress response of wheat–barley addition lines carrying chromosomes from the winter barley “Manas.”. Euphytica.

[29] Dulai S, Molnár I, Haló B, Molnár-Láng M (2010). Photosynthesis in the 7H Asakaze komugi/Manas wheat/barley addition line during salt stress. Acta Agron Hung.

[30] Darkó É, Barnabás B, Molnár-Láng M (2012). Characterization of newly developed wheat/barley introgression lines in respect of aluminium tolerance. Am J Plant Sci.

[31] Riley R, Chapman V, Johnson R (1968). The incorporation of alien disease resistance in wheat by genetic interference with the regulation of meiotic chromosome synapsis. Genet Res.

[32] Driscoll C, Sears ER. Individual addition of the chromosomes of “Imperial” rye to wheat. Agron Abst. 1971;6.

[33] Dvorak J, Knott DR (1974). Disomic and Ditelosomic Additions of Diploid *Agropyron elongatum* Chromosomes to Triticum Aestivum. Can J Genet Cytol.

[34] Miller TE, Reader SM, Chapman V. The addition of *Hordeum chilense* chromosomes to wheat. In: Induced variability in Plant Breeding. C. Pudoc ed. Wageningen: Proc Eucarpia Symp, Wageningen, The Netherlands; 1982. p. 79–81.

[35] Friebe B, Tuleen NA, Gill BS (1995). Standard karyotype of *Triticum searsii* and its relationship with other S-genome species and common wheat. Theor Appl Genet.

[36] Friebe B, Tuleen N, Jiang J, Gill BS (1993). Standard karyotype of *Triticum longissimum* and its cytogenetic relationship with *T. aestivum*. Genome.

[37] Friebe B, Jiang J, Tuleen N, Gill BS (1995). Standard karyotype of *Triticum umbellulatum* and the characterization of derived chromosome addition and translocation lines in common wheat. Theor Appl Genet.

[38] Friebe BR, Tuleen NA, Gill BS (1999). Development and identification of a complete set of *Triticum aestivum - Aegilops geniculata* chromosome addition lines. Genome.

[39] Friebe B, Qi LL, Nasuda S, Zhang P, Tuleen NA, Gill BS (2000). Development of a complete set of *Triticum aestivum-Aegilops speltoides* chromosome addition lines. Theor Appl Genet.

[40] Wang SL, Qi LL, Chen PD, Liu DJ, Friebe B, Gill BS (1999). Molecular cytogenetic identification of wheat-*Elymus tsukushiense* introgression lines. Euphytica.

[41] Suchánková P, Kubaláková M, Kovářová P, Bartoš J, Číhalíková J, Molnár-Láng M, Endo TR, Doležel J (2006). Dissection of the nuclear genome of barley by chromosome flow sorting. Theor Appl Genet.

[42] Harper LC, Cande WZ (2000). Mapping a new frontier; development of integrated cytogenetic maps in plants. Funct Integr Genomics.

[43] Mayer KFX, Martis M, Hedley PE, Simková H, Liu H, Morris JA (2011). Unlocking the barley genome by chromosomal and comparative genomics. Plant Cell.

[44] Valárik M, Bartoš J, Kovárová P, Kubaláková M, de Jong JH, Dolezel J (2004). High-resolution FISH on super-stretched flow-sorted plant chromosomes. Plant J.

[45] Bartoš J, Paux E, Kofler R, Havránková M, Kopecký D, Suchánková P, Šafář J, Šimková H, Town CD, Lelley T, Feuillet C, Doležel J (2008). A first survey of the rye (*Secale cereale*) genome composition through BAC end sequencing of the short arm of chromosome 1R. BMC Plant Biol.

[46] Tiwari VK, Wang S, Sehgal S, Vrána J, Friebe B, Kubaláková M, Chhuneja P, Doležel J, Akhunov E, Kalia B, Sabir J, Gill BS (2014). SNP Discovery for mapping alien introgressions in wheat. BMC Genomics.

[47] Szakács E, Molnár-Láng M (2010). Identification of new winter wheat - winter barley addition lines (6HS and 7H) using fluorescence in situ hybridization and the stability of the whole “Martonvásári 9 kr1” - “Igri” addition set. Genome.

[48] Yasui H, Iwata N (1998). Cytogenetics of ditelosomic alien addition lines in rice (*Oryza sativa* L.) each carrying an extra pair of telocentric chromosomes of *O. punctata* Kotschy. J Fac Agr - Kyushu Univ (Japan).

[49] Molnár-Láng M, Türkösi E, Farkas A, Cseh A, Kruppa K, Icsó D, Rakszegi M, Szakács É, Hoffmann B, Linc G. Evaluation of flowering time, ß-glucan content and tillering of wheat/barley introgression lines. In: Cereals for Food, Feed and Fuel, Challenge for Global Improvement: Eucarpia Cereals Section - ITMI Joint Conference, Book of Abstract. 359 p. Lohwasser U, Börner A.eds. Wernigerode, Germany; 2014:60.

[50] Cockram J, Jones H, Leigh FJ, O’Sullivan D, Powell W, Laurie DA, Greenland AJ (2007). Control of flowering time in temperate cereals: genes, domestication, and sustainable productivity. J Exp Bot.

[51] Turner A, Beales J, Faure S, Dunford RP, Laurie DA (2005). The pseudo-response regulator *Ppd-H1* provides adaptation to photoperiod in barley. Science.

[52] Pourkheirandish M, Komatsuda T (2007). The Importance of Barley Genetics and Domestication in a Global Perspective. Ann Bot.

[53] Laurie DA, Pratchett N, Bezant JH, Snape JW (1995). RFLP mapping of five major genes and eight quantitative trait loci controlling flowering time in a winter × spring barley (*Hordeum vulgare L.*) cross. Genome.

[54] Sameri M, Pourkheirandish M, Chen G, Tonooka T, Komatsuda T. Detection of photoperiod responsive and nonresponsive flowering time QTL in barley*.* Breeding Science. 2011;61(2):183–8.

[55] Boyd W, Li CD, Grime C, Cakir M, Potipibool S, Kaveeta L (2003). Conventional and molecular genetic analysis of factors contributing to variation in the timing of heading among spring barley (Hordeum vulgare L.) genotypes grown over a mild winter growing season. Crop Past Sci.

[56] Chang S-B, de Jong H (2005). Production of alien chromosome additions and their utility in plant genetics. Cytogenet Genome Res..

[57] Cseh A, Kruppa K, Molnár I, Rakszegi M, Doležel J, Molnár-Láng M (2011). Characterization of a new 4BS.7HL wheat–barley translocation line using GISH, FISH, and SSR markers and its effect on the β-glucan content of wheat. Genome.

[58] Cseh A, Türkösi E, King J, King I, Molnár-Láng M. Development of new winter wheat-winter barley 7BS.7HL Robertsonian translocation line. London: Plant Genomics Congress; 2015. pp. 30. Available online at http://www.globalengage.co.uk/ngs/posters15.pdf.

