# Supplementary material for: Addition of Manas barley chromosome arms to the hexaploid wheat genome
Source: BMC Genet. 2016 Jun 21;17:87. doi: 10.1186/s12863-016-0393-2 (PMC4915093; doi:10.1186/s12863-016-0393-2)
Supplement: Additional file 1: — STS and SSR markers used in the present study, the primer sequences, the annealing temperature and the size of PCR products. (DOCX 16 kb) [file 12863_2016_393_MOESM1_ESM.docx]

**Additional file 1** **STS and SSR markers used in the present study,** the primer sequences, the annealing temperature and the size of PCR products.

| **Chromosome**  **arm** | **Marker ^a^** | **Forward primer** | **Reverse primer** | **T annealing (^o^C)** | **Size (bp)** |
| --- | --- | --- | --- | --- | --- |
| 2HS | HvCSLF4 | CCGTCGGGCTCGTGTATGTC | TTGCAGTGACTCTGGCTGTACTTG | 67 | 160 |
| 2HL | Bmag0125 | AATTAGCGAGAACAAAATCAC | AGATAACGATGCACCACC | 55 | 117 |
| 3HS | HvLTPPB | TGCTGAGACGCTGAGTACGTTG | CAAACTCACGATTCCTCTCAAAG | 55 | 195, 207, 220 |
| 3HL | HvM60 | CAATGATGCGGTGAACTTTG | CCTCGGATCTATGGGTCCTT | 55 | 110, 113, 121 |
| 4HS | HvM40 | CGATTCCCCTTTTCCCAC | ATTCTCCGCCGTCCACTC | 55 | 136, 141 |
| 4HL | HvM67 | GTCGGGCTCCATTGCTCT | CCGGTACCCAGTGACGAC | 55 | 124 |
| 6HS | Bmac0316 | ATGGTAGAGGTCCCAACTG | ATCACTGCTGTGCCTAGC | 55 | 148 |
| 6HL | EBmac0806 | ACTAAGTCCTTTCACGAGGA | GTGTGTAGTAGGTGGGTACTTG | 55 | 153 |
| 7HS | Bmac0031 | AGAGAAAGAGAAATGTCACCA | ATACATCCATGTGAGGGC | 60 | 158 |
| 7HL | HvCSLF6 | TGGGCATTCACCTTCGTCAT | TGTCCGGGCAAACTCATCAA | 64 | 150 |

^a^ For further details, see http://bioinf.scri.ac.uk/ssr/ssr_table.html
