# Supplementary material for: Addition of Manas barley chromosome arms to the hexaploid wheat genome
Source: BMC Genet. 2016 Jun 21;17:87. doi: 10.1186/s12863-016-0393-2 (PMC4915093; doi:10.1186/s12863-016-0393-2)
Supplement: Additional file 2: — Morphological traits of the Asakaze/Manas wheat-barley ditelosomic addition lines, the parental wheat cultivars and Asakaze/Manas disomic addition lines in experiments carried out in the Martonvásár phytotron between December 2013 and March 2014. #: significantly different from Asakaze, ##: significantly different from Chinese Spring, ###: significantly different from Asakaze and Chinese Spring at P = 0.05. (DOCX 17 kb) [file 12863_2016_393_MOESM2_ESM.docx]

**Additional file 2 Morphological traits of the Asakaze/Manas wheat-barley ditelosomic addition lines**, the parental wheat cultivars and Asakaze/Manas disomic addition lines in experiments carried out **in the Martonvásár phytotron** between December 2013 and March 2014. ^#^: significantly different from Asakaze, ^##^: significantly different from Chinese Spring, ^###^: significantly different from Asakaze and Chinese Spring at P = 0.05

| **Phytotron 2013** | **Plant height (cm)** | **Tillering** | **Length of the main spike (cm)** | **Seeds/plant** | **Seeds/main spike** |
| --- | --- | --- | --- | --- | --- |
| **2HS** | **75.90^##^±6.06** | **3.30^#^±0.48** | **7.35^###^±0.74** | **104.30^#^±21.75** | **40.80^##^±5.51** |
| **2HL** | **76.20^##^±3.52** | **3.30^#^±0.48** | **6.90^###^±0.39** | **102.30^#^±17.04** | **36.80^###^±6.59** |
| **3HS** | **79.50^###^±5.40** | **3.20^#^±0.63** | **7.95^###^±0.43** | **97.40^#^±13.30** | **39.40^##^±9.44** |
| **3HL** | **81.90^#^±3.35** | **3.00^#^±0.82** | **5.87^#^±0.57** | **78.30^##^±19.77** | **33.50^###^±5.85** |
| **4HS** | **87.80^#^±4.21** | **2.40^##^±0.51** | **7.40^###^±0.56** | **111.10^#^±12.48** | **54.70^###^±7.19** |
| **4HL** | **55.10^###^±3.51** | **2.40^##^±0.84** | **7.90^###^±1.04** | **101.90^#^±20.35** | **52.30±14.02** |
| **6HS** | **72.70^##^±5.81** | **3.90^###^±0.74** | **10.40^##^±1.45** | **110.40^#^±36.07** | **45.50±13.25** |
| **6HL** | **82.90^#^±9.24** | **2.90^#^±0.56** | **8.50^###^±0.57** | **85.90^##^±13.06** | **37.20^###^±7.23** |
| **7HS** | **80.50±12.85** | **2.90^#^±0.87** | **10.00^##^±1.88** | **61.50^##^±40.06** | **26.90^###^±17.14** |
| **7HL** | **71.80^##^±8.97** | **4.10^###^±0.73** | **6.35^#^±0.88** | **48.10^###^±26.92** | **18.10^###^±9.07** |
| **2H** | **67.60±7.89** | **4.20±0.63** | **7.20±0.53** | **66.30±32.09** | **31.30±9.50** |
| **3H** | **75.90±11.52** | **3.30±0.48** | **8.40±1.46** | **97.80±22.81** | **34.90±2.60** |
| **4H** | **78.75±2.06** | **3.00±0.00** | **8.75±0.64** | **80.75±52.97** | **21.91±14.36** |
| **6H** | **78.60±4.00** | **3.20±0.63** | **8.95±0.59** | **75.20±15.10** | **29.80±6.12** |
| **7H** | **67.50±5.14** | **3.20±0.78** | **8.50±1.00** | **59.20±22.04** | **21.40±8.08** |
| **Asakaze** | **74.60±4.52** | **2.00±0.47** | **9.80±0.54** | **82.20±13.98** | **45.60±4.78** |
| **Chinese Spring** | **85.30±5.57** | **3.10±0.31** | **6.35±0.47** | **111.40±10.81** | **47.30±3.59** |
| **Manas** | **52.30±2.05** | **3.40±0.51** | **8.80±0.25** | **130.00±10.81** | **52.00±10.37** |
