# Supplementary material for: Addition of Manas barley chromosome arms to the hexaploid wheat genome
Source: BMC Genet. 2016 Jun 21;17:87. doi: 10.1186/s12863-016-0393-2 (PMC4915093; doi:10.1186/s12863-016-0393-2)
Supplement: Additional file 3: — Morphological traits of the Asakaze/Manas wheat-barley ditelosomic adittion lines, the parental wheat cultivars and Asakaze/Manas disomic addition lines in the Martonvásár nursery during the 2014–2015 growing season. #: significantly different from Asakaze, ##: significantly different from Chinese Spring, ###: significantly different from Asakaze and Chinese Spring at P = 0.05. (DOCX 19 kb) [file 12863_2016_393_MOESM3_ESM.docx]

**Additional file 3 Morphological traits of the Asakaze/Manas wheat-barley ditelosomic adittion lines**, the parental wheat cultivars and Asakaze/Manas disomic addition lines **in the Martonvásár nursery** during the 2014-2015 growing season. ^#^: significantly different from Asakaze, ^##^: significantly different from Chinese Spring, ^###^: significantly different from Asakaze and Chinese Spring at P = 0.05

| **Tükrös 2015** | **Plant height (cm)** | **Tillering** | **Length of the main spike (cm)** | **Seeds/plant** | **Seeds/main spike** | **1000-kernel weight (g)** |
| --- | --- | --- | --- | --- | --- | --- |
| **2HS** | **83.10** | **6.20±2.04** | **7.15^#^±0.82** | **232.40±104.19** | **44.7±11.38** | **28.90** |
| **2HL** | **89.10±9.58** | **5.90^#^±1.73** | **7.75^##^±0.95** | **237.70±95.39** | **51.70±11.62** | **23.80** |
| **3HS** | **96.10^###^±6.85** | **6.70±1.25** | **8.90^##^±1.26** | **267.90±95.00** | **49.00±8.33** | **29.80** |
| **3HL** | **98.80^###^±5.03** | **8.20±2.14** | **6.85^#^±0.94** | **288.00±120.47** | **41.90±11.82** | **34.30** |
| **4HS** | **106.40^###^±6.20** | **5.40^#^±1.17** | **7.20^#^±0.82** | **269.50±99.36 ** | **56.50^#^±14.65** | **32.50** |
| **4HL** | **79.00^###^±8.64** | **6.40±1.83** | **8.15^##^±0.91** | **365.40±151.17** | **70.30^###^±10.57** | **28.90** |
| **6HS** | **102.10^###^±4.63** | **5.70^#^±1.83** | **8.90^##^±1.13** | **253.50±91.87** | **54.50^#^±8.63** | **33.70** |
| **6HL** | **112.90^###^±7.17** | **5.80^#^±2.04** | **9.05^##^±1.06** | **264.30±105.49** | **50.20±10.11** | **37.20** |
| **7HS** | **100.50^###^±6.22** | **7.20±2.34** | **9.80^###^±1.20** | **312.20±126.77** | **53.10^#^±11.63** | **33.50** |
| **7HL** | **96.20^#^±9.46** | **5.80^#^±1.32** | **6.30^#^±0.88** | **191.90^###^±54.67** | **37.40^###^±8.23** | **27.40** |
| **2H** | **99.40** | **7.60±3.02** | **7.90±0.93** | **317.70±161.07** | **47.10±9.08** | **36.10** |
| **3H** | **105.70±5.69** | **7.50±1.90** | **9.30±1.25** | **422.50±160.70** | **59.20±16.93** | **35.40.** |
| **4H** | **93.70** | **5.70±2.11** | **8.35** | **363.00±151.64** | **71.11±5.20** | **30.00** |
| **6H** | **91.60±5.81** | **7.80±2.82** | **8.80±0.67** | **351.60±143.12** | **55.10±4.25** | **32.60** |
| **7H** | **79.70±4.05** | **8.80±2.14** | **8.40±0.81** | **283.40±122.85** | **38.30±9.36** | **29.40** |
| **Asakaze** | **88.40±3.10** | **7.80±1.93** | **8.40±0.70** | **279.10±97.90** | **44.00±4.64** | **32.40** |
| **Chinese Spring** | **89.10±4.90** | **7.00±1.70** | **6.75** | **313.40±117.05** | **47.40±8.22** | **32.30** |
| **Manas** | **82.30±4.11** | **8.10±1.73** | **7.25±0.86** | **355.50±121.56** | **52.20±10.56** | **42.10** |
