# Supplementary material for: Addition of Manas barley chromosome arms to the hexaploid wheat genome
Source: BMC Genet. 2016 Jun 21;17:87. doi: 10.1186/s12863-016-0393-2 (PMC4915093; doi:10.1186/s12863-016-0393-2)
Supplement: Additional file 4: — Effect of salt stress on the germination properties of seedlings of Asakaze/Manas ditelosomic addition lines, the wheat parent Asakaze and the barley parent Manas. The data are mean values ± standard deviation for each treatment. Significant differences were determined using Tukey’s post hoc test and different letters indicate significant differences between the genotypes and treatments at the P ≤ 0.05 level. “-” indicates that the drastic reduction caused by salt treatment prevented statistical analysis and the comparison of the genotypes. (DOC 117 kb) [file 12863_2016_393_MOESM4_ESM.doc]

|  |  | **A** | **2HS** | **2HL** | **3HS** | **3HL** | **4HS** | **4HL** | **6HS** | **6HL** | **7HS** | **7HL** | **Manas** |
| --- | --- | --- | --- | --- | --- | --- | --- | --- | --- | --- | --- | --- | --- |
| Germination% | **Control** | 100 | 100 | 100 | 100 | 100 | 100 | 100 | 100 | 100 | 100 | 100 | 100 |
|  |  | ±0  a | ±0  a | ±0  a | ±0  a | ±0  a | ±0  a | ±0  a | ±0  a | ±0  a | ±0  a | ±0  a | ±0  a |
|  | **NaCl**  **100mM** | 100 | 100 | 100 | 100 | 100 | 100 | 100 | 94.4 | 97.3 | 94.3 | 100 | 97.2 |
|  |  | ±0  a | ±0  a | ±0  a | ±0  a | ±0  a | ±0  a | ±0  a | ±4.72  a | ±4.86  ab | ±4.71  b | ±0  a | ±4.86  ab |
|  | **NaCl**  **200mM** | 86.4 | 91.4 | 100 | 74.3 | 89.5 | 85.7 | 72.9 | 68 | 56.7 | 58.4 | 92.3 | 94.28 |
|  |  | ±4.32  bc | ±4.57  b | ±0  a | ±3.72  de | ±4.48  bc | ±4.29  bc | ±3.65  de | ±3.41  ef | ±2.84  g | ±2.64  g | ±4.62  bc | ±4.71  b |
|  | **NaCl**  **250mM** | 77.3 | 45.7 | 89.2 | 60.2 | 70.3 | 64.7 | 51.35 | 43.3 | 50.55 | 52.9 | 85.66 | 87.5 |
|  |  | ±3.86  d | ±2.28  hi | ±4.45  bc | ±3  g | ±3.51  def | ±3.23  fg | ±2.57  h | ±2.17  i | ±2.53  h | ±2.92  h | ±4.71  c | ±4.37  bc |
|  |  |  |  |  |  |  |  |  |  |  |  |  |  |
| Root length (cm) | **Control** | 5.78 | 6.18 | 6.43 | 6.14 | 6.28 | 6.63 | 6.09 | 4.98 | 5.22 | 5.76 | 5.46 | 5.07 |
|  |  | ±0.346  b | ±0.432  ab | ±0.332  a | 0.368  ab | 0.251  ab | 0.298  a | 0.304  ab | 0.199  c | 0.313  bc | 0.403  b | 0.328  bc | 0.304  c |
|  | **NaCl**  **100mM** | 5.083 | 5.335 | 5.842 | 5.494 | 5.195 | 5.623 | 4.693 | 4.172 | 4.041 | 4.722 | 4.974 | 4.483 |
|  |  | ±0.213  c | ±0.213  bc | ±0.204  b | ±0.374  bc | ±0.239  bc | ±0.337  b | ±0.282  c | ±0.279  d | ±0.323  d | ±0.283  cd | ±0.209  c | ±0.197  d |
|  | **NaCl**  **200mM** | 1.902 | 1.189 | 2.042 | 1.544 | 1.631 | 2.158 | 1.599 | 0.862 | 1.013 | 0.877 | 3.427 | 3.392 |
|  |  | ±0.171  f | ±0.107  h | ±0.180  f | ±0.139  g | ±0.147  fg | ±0.194  f | ±0.144  g | ±0.077  i | ±0.091  i | ±0.079  i | ±0.223  e | ±0.237  e |
|  | **NaCl**  **250mM** | 0.882 | 0.432 | 0.885 | 0.943 | 0.651 | 0.93 | 0.415 | 0.383 | 0.311 | 0.375 | 1.675 | 1.89 |
|  |  | ±0.106  i | ±0.052  - | ±0.106  i | ±0.113  i | ±0.078  - | ±0.112  i | ±0.050  - | ±0.046  - | ±0.037  - | ±0.045  - | ±0.201  fg | ±0.227  fg |
|  |  |  |  |  |  |  |  |  |  |  |  |  |  |
| Shoot length (cm) | **Control** | 4.03 | 3.96 | 4.493 | 3.583 | 4.075 | 4.345 | 3.385 | 3.68 | 3.73 | 3.47 | 3.435 | 4.02 |
|  |  | ±0.242  b | ±0.238  b | ±0.270  a | ±0.215  c | ±0.245  ab | ±0.261  a | ±0.203  c | ±0.221  bc | ±0.224  bc | ±0.208  c | ±0.206  c | ±0.241  ab |
|  | **NaCl**  **100mM** | 2.672 | 2.564 | 3.633 | 1.914 | 2.6141 | 3.014 | 1.669 | 2.332 | 2.278 | 1.875 | 2.527 | 3.524 |
|  |  | ±0.112  e | ±0.108  e | ±0.152  c | ±0.108  g | ±0.110  e | ±0.137  d | ±0.107  g | ±0.098  f | ±0.096  f | ±0.079  g | ±0.106  ef | ±0.148  c |
|  | **NaCl**  **200mM** | 0.253 | 0.122 | 0.186 | 0.136 | 0.138 | 0.193 | 0.115 | 0.103 | 0.122 | 0.106 | 1.114 | 1.524 |
|  |  | ±0.023  - | ±0.011  - | ±0.017  - | ±0.012  - | ±0.012  - | ±0.017  - | ±0.010  - | ±0.009  - | ±0.011  - | ±0.010  - | ±0.100  h | ±0.137  gh |
|  | **NaCl**  **250mM** | 0.117 | 0.103 | 0.119 | 0.117 | 0.113 | 0.119 | 0.117 | 0.075 | 0.102 | 0.114 | 0.117 | 0.809 |
|  |  | ±0.014  - | ±0.012  - | ±0.014  - | ±0.014  - | ±0.014  - | ±0.014  - | ±0.014  - | ±0.009  - | ±0.012  - | ±0.014  - | ±0.014  - | ±0.097  i |
|  |  |  |  |  |  |  |  |  |  |  |  |  |  |
| Root weight  (mg / seedling) | **Control** | 39.9 | 39.2 | 41.2 | 39.1 | 40.8 | 49.5 | 38.9 | 33.4 | 32.1 | 35.0 | 37.4 | 36.2 |
|  |  | ±2.39  bc | ±1.96  bc | ±2.14  bc | ±2.11  bc | ±2.65  bc | ±2.92  a | ±2.29  c | ±1.82  d | ±1.76  de | ±1.68  d | ±2.02  cd | ±2.06  cd |
|  | **NaCl**  **100mM** | 29.9 | 30.4 | 34.8 | 35.8 | 37.0 | 43.0 | 34.4 | 27.7 | 24.0 | 30.4 | 32.7 | 31.5 |
|  |  | ±1.79  ef | ±1.58  ef | ±1.62  d | ±1.79  cd | ±1.93  cd | ±2.10  b | ±1.99  d | ±1.81  f | ±1.73  g | ±1.79  ef | ±2.06  de | ±1.76  e |
|  | **NaCl**  **200mM** | 9.41 | 4.77 | 8.30 | 7.42 | 7.36 | 5.72 | 4.50 | 7.14 | 2.93 | 3.23 | 16.17 | 20.04 |
|  |  | ±0.695  j | ±0.510  k | ±0.539  j | ±0.462  j | ±0.486  j | ±0.486  k | ±0.337  k | ±0.455  kj | ±0.396  l | ±0.333  l | ±1.37  i | ±1.49  h |
|  | **NaCl**  **250mM** | 2.0 | 0.52 | 0.54 | 0.53 | 1.48 | 1.53 | 0.52 | 0.51 | 0.53 | 0.52 | 7.13 | 9.6 |
|  |  | ±0.5  m | ±0.23  - | ±0.25  - | ±0.325  - | ±0.32  - | ±0.24  - | ±0.26  - | ±0.27  - | ±0.24  - | ±0.25  - | ±1.06  j | ±1.44  j |
|  |  |  |  |  |  |  |  |  |  |  |  |  |  |
| Shoot weight  (mg / seedling) | **Control** | 33.2 | 35.8 | 35.2 | 38.04 | 40.3 | 43.8 | 35.6 | 37.6 | 36.7 | 30.6 | 35.6 | 46.6 |
|  |  | ±2.43  cd | ±2.68  bc | ±2.30  c | ±2.43  bc | ±2.62  b | ±2.58  ab | ±2.10  c | ±2.65  bc | ±2.45  bc | ±2.66  cd | ±2.63  cd | ±2.66  a |
|  | **NaCl**  **100mM** | 24.8 | 21.73 | 29.09 | 20.27 | 29.41 | 37.6 | 25.66 | 28.01 | 24.34 | 20.45 | 32.18 | 43.6 |
|  |  | ±2.20  ef | ±1.88  f | ±1.97  d | ±2.05  f | ±2.18  d | ±2.2  bc | ±2.12  e | ±1.85  de | ±1.75  ef | ±1.97  f | ±2.38  d | ±2.18  ab |
|  | **NaCl**  **200mM** | 1.21 | 1.13 | 1.42 | 1.24 | 1.34 | 1.53 | 1.04 | 1.22 | 1.02 | 0.82 | 6.75 | 9.20 |
|  |  | ±0.353  - | ±0.295  - | ±0.401  - | ±0.255  - | ±0.345  - | ±0.298  - | ±0.275  - | ±0.306  - | ±0.238  - | ±0.157  - | ±0.641  g | ±1.128  g |
|  | **NaCl**  **250mM** | 0.51 | 0.49 | 0.51 | 0.50 | 0.45 | 0.55 | 0.43 | 0.45 | 0.52 | 0.51 | 0.55 | 0.56 |
|  |  | ±0.125  - | ±0.122  - | ±0.127  - | ±0.125  - | ±0.112  - | ±0.137  - | ±0.129  - | ±0.115  - | ±0.134  - | ±0.127  - | ±0.082  - | ±0.075  - |

Additional file 4 **Effect of salt stress on the germination properties of seedlings of Asakaze/Manas ditelosomic addition lines**, the wheat parent Asakaze and the barley parent Manas. The data are mean values ± standard deviation for each treatment. Significant differences were determined using Tukey’s post hoc test and different letters indicate significant differences between the genotypes and treatments at the P ≤ 0.05 level. “-“ indicates that the drastic reduction caused by salt treatment prevented statistical analysis and the comparison of the genotypes.
